# Supplementary material for: Hydrodynamic and Electrophoretic Properties of Trastuzumab/HER2 Extracellular Domain Complexes as Revealed by Experimental Techniques and Computational Simulations
Source: Int J Mol Sci. 2019 Mar 1;20(5):1076. doi: 10.3390/ijms20051076 (PMC6429128; doi:10.3390/ijms20051076)
Supplement: Supplementary file 1 [file ijms-20-01076-s001.pdf]

SUPPORTING INFORMATION

We have studied the hydrodynamic properties of TZM at different concentrations in order to test the possibility of aggregation. As it can be observed in Figures S1 and S2, no signature of aggregates is observed in the samples between 0.5 – 1.5 mg/mL. The results shown correspond to a temperature of 293 K. Variations in temperature between 12 and 35 °C give rise to the same results.

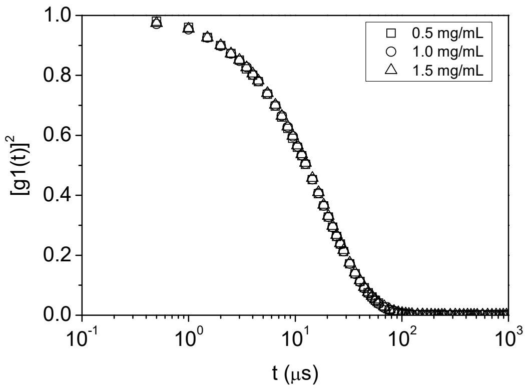

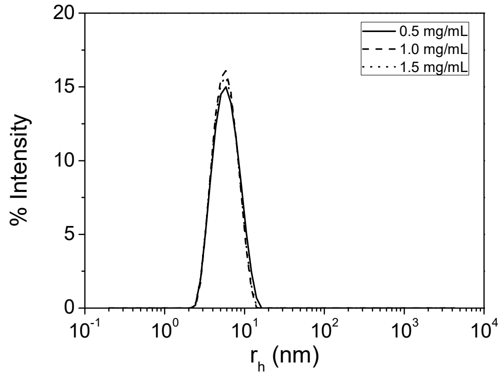

Figures S1 (left). Autocorrelation function obtained for TZM at 20 °C in different sample concentration. **Figure S2 (right).** Derived intensity size distributions obtained from the different concentrations.

The stability of the TZM samples makes possible to determine the molecular weight and the virial coefficient from batch static light scattering (SLS) experiments in the same range of concentrations. SLS data were obtained using also the Zetasizer Nano ZS apparatus. The intensity measurements for different sample concentrations in the dilute regime for solutions were obtained at T = 293 K. The results were fitted by linear regression to the Zimm equation:

$$\frac{Kc}{R_\theta} = \left[\frac{1}{M_w} + 2B_{22}c\right]\frac{1}{P(\theta)}$$

where M_w is the weight average molecular weight of the solute, B_{22} the second virial coefficient, and c its concentration in g·mL^{-1}. K is the optical constant given by:

$$K = \frac{2\pi^2 n_0}{N_a \lambda_0^4}\left(\frac{dn}{dc}\right)^2$$

where N_a is the Avogadro number, and dn/dc is the specific refractive index increment of the solution, which was set at 0.185 mg·mL^{-1}, the corresponding value for proteins, and the laser wavelength, λ_0 = 633 nm. The Rayleigh ratio, R_θ, was calculated by subtracting the solvent intensity from the solution intensity ($I_A = I_{Solution} - I_{Solvent}$) using toluene as the standard (I_T) for which $R_T = 1.41\times10^{-5}$ cm^{-1} and λ_0 = 633 nm:

$$R_\theta = \frac{I_A n_0^2}{I_T n_T^2} R_T$$

where n_0 and n_T are the refractive index of the solvent and toluene, respectively. Each final $R_\theta$data point was based on averaging not less than 10 statistically consistent measurements. Finally, P(θ) is the shape factor, which embodies the angular dependence of the sample scattering intensity that occurs when the particles are big enough to accommodate multiple photon scattering. When the particles in solution are much smaller than the wavelength of the incident light, multiple photon scattering is avoided. Under these conditions, the angular dependence of the scattering intensity is lost and P(θ) takes the value of 1. The use of this simple approximation assumes that the third and higher virial terms contribute only negligibly to the measurements; this assumption can be considered valid under the low protein concentrations used in our experiments. In Figure S3 we can observe the Debye plot obteined from such experiments. The intercept of the straight lines is the reciprocal value of the weight average molecular weight, $1/M_w$. From the value of the intercept, M_w is obtained as 137 ± 5 kDa, which nicely agrees with the obtained from SEC experiments. Additionally, a positive value of the virial coefficient, B_{22}, is obtained from the slope of the line, suggesting a great stability of TZM in the conditions used in this work.

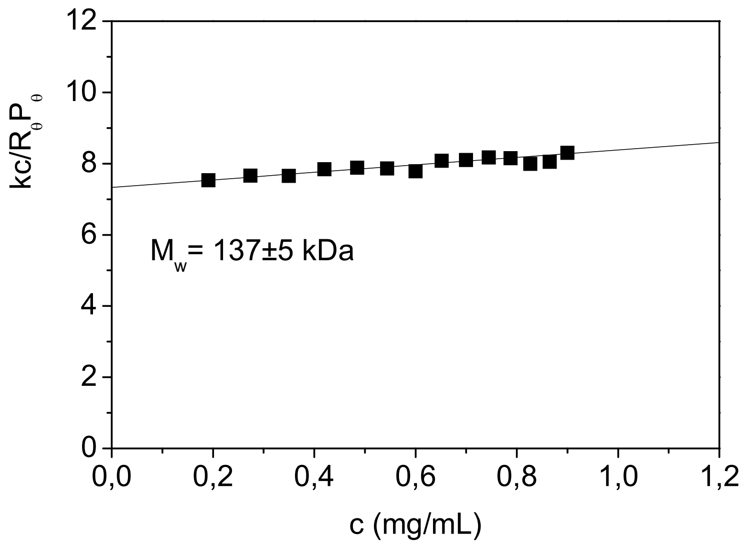

Figure S3. Debye plot obtained for TZM samples by static light scattering.
